# Detection of acute traumatic coagulopathy and massive transfusion requirements by means of rotational thromboelastometry: an international prospective validation study

**DOI:** 10.1186/s13054-015-0823-y

**Published:** 2015-03-23

**Authors:** Jostein S Hagemo, Sarah C Christiaans, Simon J Stanworth, Karim Brohi, Pär I Johansson, J Carel Goslings, Paal A Naess, Christine Gaarder

**Affiliations:** Department of Research, Norwegian Air Ambulance Foundation, Postboks 39 1441, Drøbak, Norway; Department of Anaesthesiology, Oslo University Hospital, Rikshospitalet, Postboks 4950, Nydalen, 0424 Oslo, Norway; Trauma Unit, Department of Surgery, Academic Medical Centre, University of Amsterdam, Meibergdreef 9, 1105 AZ Amsterdam, Netherlands; Department of Anesthesiology, University of Alabama at Birmingham, 1720 2nd Avenue S, Birmingham, AL 35233 USA; NHS Blood & Transplant and Oxford University Hospitals NHS Trust, John Radcliffe Hospital, Headley Way, Headington, Oxford, OX3 9DU UK; Trauma Sciences, Blizard Institute, Barts and the London School of Medicine and Dentistry, Queen Mary University of London, The Blizard Building, 4 Newark Street, London, E1 2AT UK; Section for Transfusion Medicine, Capital Region Blood Bank, Rigshospitalet, University of Copenhagen, Klinisk Immunologisk Afdeling Rigshospitalet, Blegdamsvej 9, 2100 København Ø, Copenhagen, Denmark; Department of Surgery, University of Texas Medical School, 6414 Fannin Street, Houston, TX 77030 USA; Department of Traumatology, Oslo University Hospital, Ullevål, Postboks 4950 Nydalen 0424, Oslo, Norway

## Abstract

**Introduction:**

The purpose of this study was to re-evaluate the findings of a smaller cohort study on the functional definition and characteristics of acute traumatic coagulopathy (ATC). We also aimed to identify the threshold values for the most accurate identification of ATC and prediction of massive transfusion (MT) using rotational thromboelastometry (ROTEM) assays.

**Methods:**

In this prospective international multicentre cohort study, adult trauma patients who met the local criteria for full trauma team activation from four major trauma centres were included. Blood was collected on arrival to the emergency department and analyzed with laboratory international normalized ratio (INR), fibrinogen concentration and two ROTEM assays (EXTEM and FIBTEM). ATC was defined as laboratory INR >1.2. Transfusion requirements of ≥10 units of packed red blood cells within 24 hours were defined as MT. Performance of the tests were evaluated by receiver operating characteristic curves, and calculation of area under the curve (AUC). Optimal cutoff points were estimated based on Youden index.

**Results:**

In total, 808 patients were included in the study. Among the ROTEM parameters, the largest AUCs were found for the clot amplitude (CA) 5 value in both the EXTEM and FIBTEM assays. EXTEM CA5 threshold value of ≤37 mm had a detection rate of 66.3% for ATC. An EXTEM CA5 threshold value of ≤40 mm predicted MT in 72.7%. FIBTEM CA5 threshold value of ≤8 mm detected ATC in 67.5%, and a FIBTEM CA5 threshold value ≤9 mm predicted MT in 77.5%. Fibrinogen concentration ≤1.6 g/L detected ATC in 73.6% and a fibrinogen concentration ≤1.90 g/L predicted MT in 77.8%. Patients with either an EXTEM or FIBTEM CA5 below the optimum detection threshold for ATC received significantly more packed red blood cells and plasma.

**Conclusions:**

This study confirms previous findings of ROTEM CA5 as a valid marker for ATC and predictor for MT. With optimum threshold for EXTEM CA5 ≤ 40 mm and FIBTEM CA5 ≤ 9 mm, sensitivity is 72.7% and 77.5% respectively. Future investigations should evaluate the role of repeated viscoelastic testing in guiding haemostatic resuscitation in trauma.

## Introduction

Haemorrhagic shock following injury has been shown to induce coagulopathy [1-3]. Acute traumatic coagulopathy (ATC) may potentiate bleeding and is associated with multiple organ failure and increased mortality [2,4,5]. Early detection of coagulopathy is important in order to counteract the haemostatic disturbances. Standard tests such as prothrombin time (PT), activated partial thromboplastin time (aPTT), fibrinogen concentration and platelet count are widely used to guide resuscitation in trauma patients [6,7]. However, the conventional coagulation tests (CCTs) focus on selected aspects of coagulation, which may not be appropriate for ATC [8]. Full blood viscoelastic haemostatic assays (VHA), such as rotational thromboelastometry (ROTEM) and thromboelastography (TEG), may provide a more complete assessment of haemostasis and as point-of-care devices should be able to provide results in a more clinically useful time frame for targeted therapy [9-11].

In a previous prospective cohort study, the tissue factor (TF)-activated ROTEM assay (EXTEM) was used to characterize ATC and the need for transfusions [12]. This study suggested that coagulopathy could be identified using the clot amplitude five minutes after the initiation of clot build-up (CA5). Thus, the CA5 value potentially may be used as a diagnostic tool for detecting ATC and the need for massive transfusion.

The objective of our study was to re-evaluate the previous findings in a larger international multi-centre setting. Specifically, we aimed to identify the threshold values that most accurately identify ATC and the need for massive transfusion, using the EXTEM assay, as well as the platelet-inhibited FIBTEM assay.

## Methods

### Design and patient selection

This multi-centre observational cohort study was conducted as a part of the Activation of Coagulation and Inflammation in Trauma study (ACIT) 3, led by the International Trauma Research Network (INTRN) collaboration. Patients were non-consecutively recruited at four major trauma centers in three different countries: UK, Denmark and Norway. The inclusion period was from January 2007 to November 2011, thereby also including a cohort previously studied [12]. Patients 18 years or older requiring full trauma team activation were eligible for inclusion. Patients who received more than 2,000 mL of fluids before arrival or who arrived in the emergency department (ED) more than two hours from time of injury were excluded. Additional exclusion criteria comprised patients who were pregnant, had known liver failure, bleeding disorders or were taking oral anticoagulant medications other than acetyl salicylic acid.

Informed consent was obtained from participating patients or their next of kin where appropriate. The study was performed in accordance with local ethical regulations and approved by local ethical authorities as specified under acknowledgements.

### Sampling techniques and measurements

Blood samples were collected within 20 minutes of arrival in hospital. Samples for ROTEM and CCTs were collected in citrated tubes, whereas samples for blood gas analyses were collected in heparinized syringes in accordance with local routines. ROTEM assays were performed within one hour by dedicated study personnel using the ROTEM Delta (TEM; TEM International, Munich, Germany). The assays used were the EXTEM assay, where the citrated sample is recalcified before it is activated by TF, and the FIBTEM assay, where the platelet inhibitor cytochalasin D was added for platelet inhibition, to isolate the fibrin component of the clot.

The clotting time (CT) of the ROTEM trace is the time from initiation of the test to first detectable rotational resistance. Clot formation time (CFT) is the time from first detectable resistance to trace amplitude of 20 mm. The alpha angle is the angle of increase at the point where 20 mm amplitude is reached. Maximum clot firmness (MCF) is the maximum clot amplitude detected. The clot amplitude (CA) after 5 (CA5) and 10 (CA10) minutes were also recorded. Due to the fact that the FIBTEM trace rarely reaches amplitude of 20 mm, the CFT and alpha angle was omitted for the FIBTEM assays in this study.

CCTs and blood gas analyses were performed with the shortest possible delay. The CCTs included in this study were PT, fibrinogen concentration and platelet count. PT was converted to international normalized ratio (INR) in accordance with the specific reagents and device characteristics in the respective laboratories. Fibrinogen was measured by the Clauss method [13].

### Data collection and statistical analyses

Patient data on demographics, time of injury, pre-hospital fluid administration and vital signs were collected prospectively. The total amount of packed red blood cell (PRBC) and plasma units required within the first 24 hours were recorded. Mechanism of injury and Injury Severity Score (ISS) were retrieved from the respective institutional trauma registries. ATC was defined as an INR value >1.2, consistent with the previous study [12]. We defined massive transfusion (MT) as the administration of 10 or more units of PRBC within 24 hours.

Groups with ATC and need for MT were compared to normal groups by Student’s *t* test or Mann-Whitney *U* test as appropriate. Receiver operating characteristic (ROC) curves and area under the curve (AUC) were used to compare test accuracy. Optimal threshold for best sensitivity and specificity was defined using the Youden index. One-way analysis of variance (ANOVA) was used for detection of differences in transfusion requirements between groups. Statistical calculations were made using SPSS 21.0 (IBM Corp Armonk, NY, USA) and MedCalc 3.0 (MedCalc Software, Ostende, Belgium). A *P* value <0.05 was considered statistically significant. Values are given as mean (standard deviation) unless stated otherwise.

## Results

A total of 808 patients were included in this study. The patient cohort is described in Table 1. Massive transfusion was required for 49 patients (6.1%) and 89 patients (11.0%) had ATC. All ROTEM parameters and CCTs differed significantly between ATC and non-ATC groups, as well as between MT and non-MT groups (*P* <0.001). These differences were also significant in the subgroup of patients presenting with a BE < −5 mEq/L.

**Table 1:** **Descriptive statistics for the study population (n = 808)**

	**All (n = 808)**	**INR >1.2 (n = 89)**	**MT (n = 49)**
Age	38 (28)	38 (29)	41 (33)
Male gender (%)	77.4	71.9	65.3
ISS	16 (20)	33 (22)	29 (16)
Penetrating injury (%)	17.5	17.1	12.24
Base excess (mEq/ml)	−1.90 (4.90)	−8.0 (8.7)	−9.9 (7.7)
ISS >15 (%)	52.5	89.2	93.6
Base excess < −5 (%)	19.5	63.5	78.7
INR >1.2 (%)	11.0	100	51.1
Any PRBC administered (%)	31.7	76.7	100
PRBC ≥10 administered (%)	6.1	27.9	100

Age, ISS and base excess are given as median (interquartile range). INR, international normalized ratio; MT, massively transfused (≥10 PRBC); ISS, Injury Severity Score; PRBC: packed red blood cells.

Test characteristics based on previously suggested threshold values for INR (>1.2), CA5 (<35 mm), CT (>94 seconds) and alpha angle (<65°) are presented in Table 2. The detection rate for MT requirement was found to be highest for INR and EXTEM CA5 with 51.1% and 45.5%, respectively.

**Table 2:** **Test characteristics in predicting massive transfusion (≥10 units of packed red blood cells) based on previously suggested threshold values** [12 **]**

	**Detection rate**		**False positive rate**		**PPV**		**NPV**	
*INR >1.2*	51.1	(36.1-65.9)	8.8	(6.8-11.0)	27.3	(18.3-37.9)	96.7	(95.0-97.9)
*CT >94 sec*	28.9	(16.4-44.3)	8.8	(6.9-11.2)	16.5	(9.1-26.5)	95.5	(93.7-96.9)
*CA5 ≤ 35 mm*	45.5	(30.4-61.2)	16.1	(13.5-19.0)	14.4	(9.0-21.3)	96.3	(94.5-97.6)
*Alpha angle <65°*	37.2	(23.0-53.3)	12.2	(9.9-14.8)	15.1	(8.9-23.4)	96.0	(94.2-97.3)

PPV, positive predictive value; NPV, negative predictive value; INR, international normalized ratio; CT, clotting time; CA5, clotting amplitude after 5 minutes.

Table 3 summarizes test performance measured by AUC for ROTEM parameters and CCTs. All included ROTEM parameters, fibrinogen concentration, INR and platelet counts significantly predicted MT. The highest ROTEM AUC values were found for EXTEM CA5 and FIBTEM CA5, both in detecting ATC and predicting MT requirements. These AUC values did however not differ significantly from the AUC of the other ROTEM parameters. AUC for fibrinogen concentration, on the other hand, was significantly higher than any other ROTEM parameter in detecting ATC.

**Table 3:** **ROC analyses of parameters predicting acute traumatic coagulopathy (ATC) and massive transfusion (MT)**

	**ATC**		**MT**	
	***AUC***	***(95% CI)***	***AUC***	***(95% CI)***
*EXTEM CT (s)*	0.73	(0.70-0.76)	0.68	(0.65-0.71)
*EXTEM CA5 (mm)*	0.79	(0.76-0.81)	0.75	(0.72-0.78)
*EXTEM CA10 (mm)*	0.78	(0.75-0.81)	0.75	(0.72-0.78)
*EXTEM CFT (s)*	0.77	(0.74-0.80)	0.73	(0.70-0.76)
*EXTEM Alpha (°)*	0.78	(0.75-0.81)	0.73	(0.69-0-76)
*EXTEM MCF (mm)*	0.73	(0.70-0.76)	0.70	(0.67-0.73)
*FIBTEM CT (s)*	0.72	(0.68-0.75)	0.65	(0.62-0.69)
*FIBTEM CA5 (mm)*	0.80	(0.77-0.83)	0.78	(0.74-0.81)
*FIBTEM CA10 (mm)*	0.79	(0.76-0.82)	0.76	(0.73-0.79)
*FIBTEM MCF (mm)*	0.77	(0.74-0.80)	0.76	(0.73-0.79)
*Fibrinogen concentration*	0.87^*^	(0.84-0.89)	0.81	(0.78-0.83)
*INR*	N/A	N/A	0.82	(0.79-0.84)
*Platelet count*	0.74	(0.70-0.77)	0.70	(0.66-0.73)

ATC, acute traumatic coagulopathy defined as INR >1.2. MT, massive transfusion defined as 10 or more packed red blood cells. All AUCs values are statistically different from 0.5 with a *P* ≤0.001. ^*^AUC is significantly larger than the AUC of the ROTEM parameters (*P* = 0.002 for difference to FIBTEM CA5). ROC, receiver operating characteristics; AUC, area under the curve; CT, clotting time; CA5, clot amplitude after 5 minutes; CA10, clot amplitude after 10 minutes; CFT, clot formation time; MCF, maximum clot firmness; INR, international normalized ratio.

The optimal threshold value for specificity and sensitivity for EXTEM CA5 in detecting ATC was found to be ≤37 mm, and in predicting MT ≤40 mm (Table 4). The corresponding values for FIBTEM were ≤8 mm and ≤9 mm, respectively. The optimal threshold for fibrinogen concentration in detecting ATC was ≤1.61 g/L and ≤1.90 g/L in predicting MT.

**Table 4:** **Optimum thresholds and respective test accuracy parameters for predicting (a) acute traumatic coagulopathy (ATC) defined as INR >1.2 and (b) massive transfusion (MT) (defined as ≥10 units of PRBC)**

***Test parameter***	***Optimum threshold***		***Detection rate***		***False positive rate***		***PPV***		***NPV***	
**(a)**										
*EXTEM CA5*	≤37	(34-39)	66.3	(55.1-76.3)	18.8	(15.9-21.9)	29.9	(23.4-37.1)	95.2	(93.2-96.8)
*FIBTEM CA5*	≤8	(5-8)	67.5	(55.9-77.8)	20.7	(17.7-23.9)	26.9	(20.8-33.8)	95.6	(93.5-97.1)
*Fibrinogen*	≤1.61	(1.36-1.9)	73.6	(63.0-82.4)	11.5	(9.2-14.1)	45.1	(36.7-53.6)	96.3	(94.5-97.7)
*Platelet count*	≤199	(128-199)	*61.7*	(46.4-75.5)	29.9	(26.6-33.4)	11.9	(8.1-16.7)	96.5	(94.6-97.9)
**(b)**										
*EXTEM CA5*	≤40	(32-40)	72.7	(57.2-85.0)	31.3	(28.0-34.8)	12.2	(8.5-16.8)	97.7	(96.0-98.8)
*FIBTEM CA5*	≤9	(6-9)	77.5	(61.5-89.2)	32.8	(29.4-36.4)	11.4	(7.9-15.8)	98.2	(96.6-99.2)
*Fibrinogen*	≤1.90	(1.39-2.18)	77.8	(62.9-88.8)	29.7	(26.4-30.1)	14.0	(9.9-18.9)	98.1	(96.5-99.1)
*INR*	≥1.13	(1.0-1.16)	70.2	(55.1-82.7)	19.0	(16.2-22.1)	19.2	(13.6-25.9)	97.7	(96.2-98.7)
*Platelet count*	≤174	(159-182)	52.8	(41.9-63.5)	14.8	(12.2-17.7)	32.2	(24.7-40.4)	93.1	(90.8-95.0)

INR, international normalized ratio; PRBC, packed red blood cells; PPV, positive predictive value; NPV, negative predictive value; CA5, clot amplitude after 5 minutes.

With the calculated optimal thresholds for MT, detection rate with EXTEM CA5 was 72.7%, for FIBTEM CA5 77.5%, for fibrinogen concentration 77.8% and for INR 70.2%.

**Figure 1:**
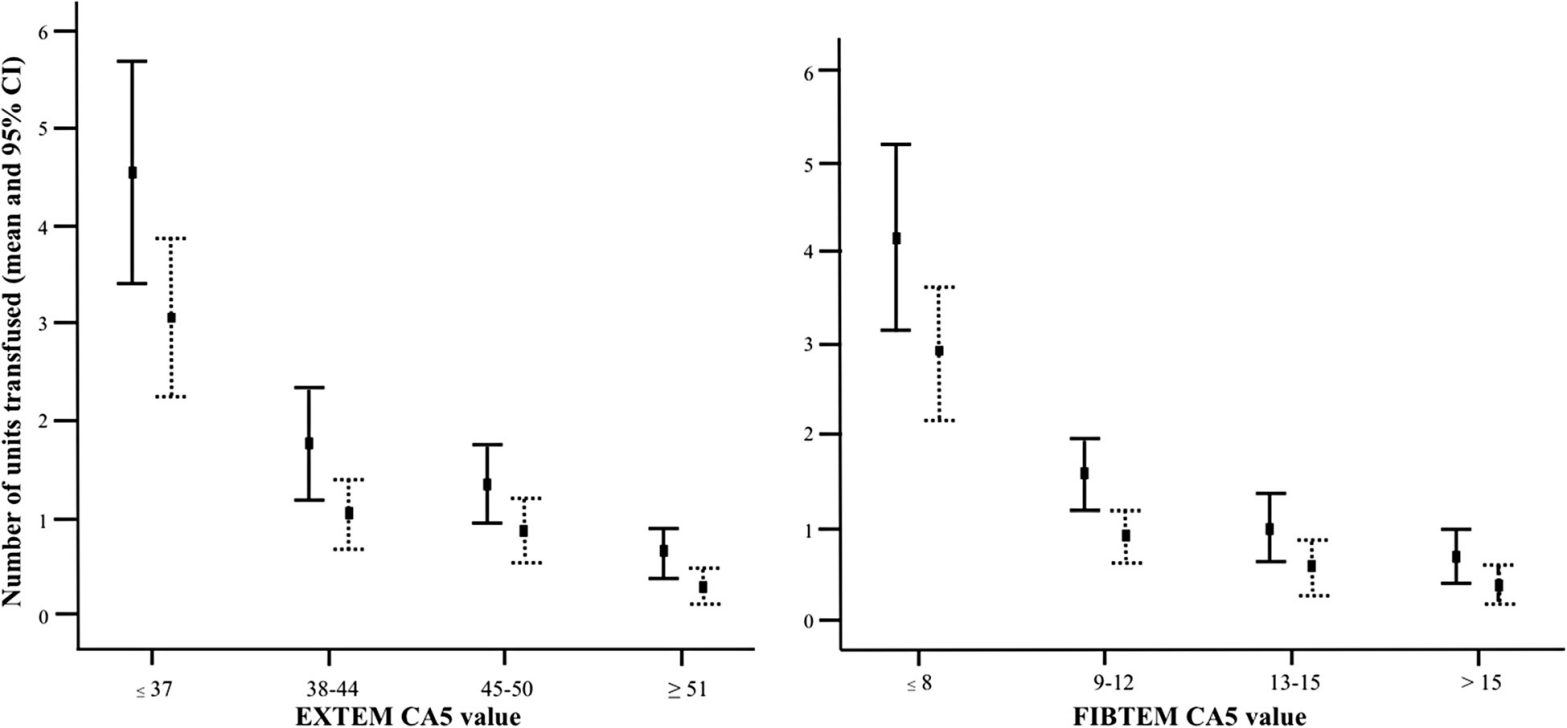
**Units transfused grouped by EXTEM CA5 (left panel) and FIBTEM CA5 (right panel).** The difference between the number of packed red blood cells (unbroken lines) and plasma units (dotted lines) in the group with CA5 below optimum threshold (≤37 mm and ≤8 mm respectively) and other groups is statistically significant. CA5, clot amplitude after 5 minutes.

The number of units PRBC and plasma transfused was significantly higher in the groups with either EXTEM CA5 or FIBTEM CA5 below the optimum threshold for ATC detection as depicted in Figure 1.

## Discussion

This study shows that the amplitude of the ROTEM assay after five minutes (EXTEM CA5) detects ATC and predicts the need for MT. The detection rate for MT of 45.5% was, however, lower in the current study compared to the predictive values previously reported (71.4%) when a threshold value of ≤35 mm was used [12]. When false positive and false negative test results were weighted equally, the threshold for best sensitivity and specificity (≤40 mm) was slightly higher in our data set than the threshold suggested by Davenport *et al*. With a threshold of ≤40 mm we found the detection rate for EXTEM CA5 to be 72.7%, comparable to the previous findings. This was, however, associated with an increased false positive rate in our data set (31.3% versus 15.3%). The reasons for the differences between our results and the results of the single-centre study by Davenport *et al*. may be due to the differences in number of massively transfused patients (11 vs. 49).

From the ROC curve analyses it appears that the platelet-inhibited assay (FIBTEM) may increase the test accuracy with respect to the need for MT. This is in accordance with the findings reported by Schöchl *et al*. [14]. In a retrospective single-centre study of 323 trauma patients, they found that the FIBTEM assay had a better overall test accuracy than the EXTEM assay. They identified the FIBTEM MCF as the parameter with the largest AUC, with detection rate similar to that of FIBTEM CA5 in our study. They identified an optimum threshold of FIBTEM MCF of ≤7 mm, with a sensitivity of 77.5%. In their study, fibrinogen concentration also had a large AUC, comparable to the best ROTEM parameter, and a sensitivity of 84.2%, with a threshold of 1.48 g/L.

Excellent correlation has previously been demonstrated between CA in platelet-inhibited ROTEM assays and fibrinogen measured by the Clauss method [15-17] Low fibrinogen concentration has been closely linked to mortality and need for MT in a number of studies [4,18,19]. In a study by Harr *et al*. [16] fibrinogen concentration was closely correlated to the clot strength (R^2^ = 0.87) in an assay similar to the FIBTEM assay (TEG Functional Fibrinogen assay). Adding fibrinogen *in vitro* increased both clot strength and the relative contribution to clot strength of fibrinogen compared to platelets. This finding is supported by animal studies, case reports and observational studies in humans demonstrating a reversal of ATC by fibrinogen concentrate [20-23]. The crucial role of fibrinogen in traumatic coagulopathy, supported by these findings, may to some extent explain why the FIBTEM assay present better test characteristics than the EXTEM assay in our study.

VHAs may benefit from several advantages compared to CCTs. Multiple repeated measurements used to evaluate the dynamic changes and to specifically direct the mode of coagulation support, has been advocated [24,25]. The ability to visualize the haemostatic process in whole blood from initiation to fibrinolysis, contrasts that of CCTs, which only assess isolated parts of the coagulation in plasma. Traditionally, the turnaround time for CCTs may be considered too long, and since the introduction of VHAs in trauma management, some researchers propose that the role of CCTs in guiding transfusion therapy is marginalized [26,27]. However, it should be noted that based on the results of several studies, INR [14,28], fibrinogen concentration [14], and haemoglobin concentration (or haematocrit) [14,29] appear to be non-inferior to VHAs when it comes to predicting MT from a single blood sample on arrival. Readily available point-of-care testing devices may, in the case of haemoglobin concentration and INR, overcome the time delay usually associated with the conventional laboratory analyses. The precision and feasibility of such diagnostics should be a target for further studies.

Limitations of our study include the fact that few patients required MT, and the confidence intervals of the test characteristics are correspondingly wide. The test results from ROTEM analyses were not blinded to clinicians in all centres and may to some extent have biased the results. In the case of such a bias this would have favoured the test performance of VHAs since they usually are available to clinicians faster than the CCTs. A survivor bias in this study cannot be excluded as some patients may have died before receiving the required amount of transfusions. This potential bias may have resulted in an underestimation of the accuracy in predicting MT in our study. Our analyses are based only on the first sample obtained shortly after arrival in the ED. The value of repeated VHA analyses to guide transfusion during the course of resuscitation was not evaluated in this study. Finally, our study is not addressing the impact of ROTEM on clinical outcomes.

## Conclusions

In conclusion, this study confirms the previous finding that the ROTEM CA5 value measured on arrival is a valid marker for ATC and predicts MT requirements. An EXTEM CA5 threshold value of ≤40 mm has a detection rate of 72.7%, whereas a FIBTEM CA5 threshold value of ≤9 mm detects MT requirements in 77.5% of cases. Fibrinogen concentration was significantly better than ROTEM assays in predicting ATC, and a fibrinogen concentration ≤1.90 g/L had a detection rate of 77.8% for MT requirement. Future studies should be directed at identifying the role of repeated VHA measurements in guiding haemostatic resuscitation in trauma.

## Key messages

- The ROTEM assay is a valid predictor of coagulopathy and MT.
- Optimal cutoff value for CA5 was found at ≤40 mm for the EXTEM assay and ≤9 mm for the FIBTEM assay.
- Using the optimal CA5 cutoff point, detection rate for massive transfusion was 72.7% and 77.5% for EXTEM and FIBTEM respectively.
- Test performance of fibrinogen concentration measured by the Clauss method was comparable to the best ROTEM parameters.

## Abbreviations

ANOVA: analysis of variance
aPTT: activated partial thromboplastin time
ATC: acute traumatic coagulopathy
AUC: area under the curve
CA: clot amplitude
CCT: conventional coagulation tests
CFT: clot formation time
CT: clotting time
ED: emergency department
INR: international normalized ratio
ISS: Injury Severity Score
MCF: maximum clot firmness
MT: massive transfusion
PRBC: packed red blood cells
PT: prothrombin time
ROC: receiver operating characteristics
ROTEM: rotational thromboelastometry
TEG: thromboelastography
TF: tissue factor
VHA: viscoelastic haemostatic assay
